# Structure-based design and classifications of small molecules regulating the circadian rhythm period

**DOI:** 10.1038/s41598-021-97962-5

**Published:** 2021-09-16

**Authors:** Seref Gul, Fatih Rahim, Safak Isin, Fatma Yilmaz, Nuri Ozturk, Metin Turkay, Ibrahim Halil Kavakli

**Affiliations:** 1 Department of Chemical and Biological Engineering, Koc University, Rumelifeneri Yolu, Sariyer, Istabul Turkey; 2 Department of Industrial Engineering, Koc University, Rumelifeneri Yolu, Sariyer, Istabul Turkey; 3 Department of Molecular Biology and Genetics, Rumelifeneri Yolu, Sariyer, Istabul Turkey; 4 Department of Molecular Biology and Genetics, Gebze Technical University, Gebze, 41400 Kocaeli Turkey

**Keywords:** Virtual drug screening, Computational models

## Abstract

Circadian rhythm is an important mechanism that controls behavior and biochemical events based on 24 h rhythmicity. Ample evidence indicates disturbance of this mechanism is associated with different diseases such as cancer, mood disorders, and familial delayed phase sleep disorder. Therefore, drug discovery studies have been initiated using high throughput screening. Recently the crystal structures of core clock proteins (CLOCK/BMAL1, Cryptochromes (CRY), Periods), responsible for generating circadian rhythm, have been solved. Availability of structures makes amenable core clock proteins to design molecules regulating their activity by using in silico approaches. In addition to that, the implementation of classification features of molecules based on their toxicity and activity will improve the accuracy of the drug discovery process. Here, we identified 171 molecules that target functional domains of a core clock protein, CRY1, using structure-based drug design methods. We experimentally determined that 115 molecules were nontoxic, and 21 molecules significantly lengthened the period of circadian rhythm in U2OS cells. We then performed a machine learning study to classify these molecules for identifying features that make them toxic and lengthen the circadian period. Decision tree classifiers (DTC) identified 13 molecular descriptors, which predict the toxicity of molecules with a mean accuracy of 79.53% using tenfold cross-validation. Gradient boosting classifiers (XGBC) identified 10 molecular descriptors that predict and increase in the circadian period length with a mean accuracy of 86.56% with tenfold cross-validation. Our results suggested that these features can be used in QSAR studies to design novel nontoxic molecules that exhibit period lengthening activity.

## Introduction

The circadian clock is a biochemical oscillator that modulates several physiologic functions such as alertness, memory, heart rate, blood pressure, and immune responses through periodic transcriptional regulation^1–5^. Additionally, genetic and epidemiologic studies have linked clock disruption with various adverse metabolic phenotypes^6^, sleep^7^ and mood disorders^8^.

At the molecular level, four core clock proteins are required to generate circadian rhythm, which are BMAL1, CLOCK, CRYPTOCHROMEs (CRYs), and PERIODs (PERs). Among these BMAL1 and CLOCK form heterodimer and bind E-box on DNA (CACGTG) and in turn, initiate transcription of clock-controlled genes (CCGs) including *Per*s and *Cry*s^9–11^. Then, PERs and CRYs accumulate in the cytosol and form a trimeric complex with casein kinase Iε/δ (CKI) and then translocate into the nucleus. Trimeric complex interacts with BMAL1/CLOCK and inhibits transcription of CCGs^12^. Period determination in the mammalian circadian clock involves the turnover rate of the CRY and PER via post-translational modifications. FBXL3 and FBXL21 mediate the degradation of CRY proteins^13,14^. A recent next-generation RNA sequencing analysis indicated that 10% of all genes and 43% of all protein-coding genes are under the control of the circadian clock in at least one tissue^15^.

Several diseases are associated with disruption of circadian rhythm at genetic level^16–20^. Studies show a broad role for the clock in normal physiology and its role in mediating pathophysiological conditions. The importance of a robust circadian clock for health is increasingly recognized, and therefore, the identification of molecules that modulate circadian clocks became a hot topic^21–28^. High-throughput screening is currently instrumental for identifying the molecules that affect the circadian clock.

Structure-based drug discovery methods have advantageous in terms of saving time and reducing cost. The addition of classification methods to the drug discovery pipeline will eliminate inappropriate molecules such as toxic and inactive. The feasibility of such implementation between them is shown in quantitative structure–activity relationships (QSAR) models for many targets^29–37^. Given the importance of circadian rhythm in human health, with the recent reports of resolved crystal structures of core clock proteins and their interacting partners (CRY-FBXL3 (pdbID: 4K0R)^38^, BMAL1-CLOCK (pdbID:4F3L)^39^, and CRY-PER (pdbID:4U8H)^40^, now it is possible to perform in silico screening to find small molecules targeting core clock proteins. A recent study reports the discovery of a molecule that regulates CLOCK and BMAL1 interaction using the structure-based approach^22^.

In this study, we performed in silico screening using CRY1 crystal structure (ID: 4K0R) to find molecules that regulate circadian rhythm in U2OS cell line. We experimentally tested 171 molecules in terms of toxicity and activity. The 56 molecules were found to be toxic, and 115 molecules were nontoxic to the cell, and 22 molecules significantly lengthened the period of the circadian rhythm. To identify molecular features, using machine learning, we used 171 molecules and our result showed that 11 features among the available 1538 were the best to predict the toxicity of the molecules. Similarly, we determined 10 molecular descriptors that explain the period change in circadian rhythm. Our results suggest that these molecular descriptors can be used in QSAR studies for the identification of nontoxic and circadian period lengthener molecules using big libraries that can be used in various CRY1 related disorders.

## Material and methods

### Molecular dynamics simulation

Mouse-CRY1 (mCRY1) (PDB ID: 4K0R) which is 93% identical to human CRY1 protein was retrieved from the protein databank. The structure was solvated in a rectangular box with TIP3P water molecules with the size of 7.25 × 10^5^Å^3^ and neutralized with counterions using the NAMD (v. 2.6)^41^ program packages. Then the system was minimized using the conjugate gradient method and kept the backbone atoms of the protein frozen. Then further minimization steps with relaxed backbone atoms were carried out. The system was heated up to physiological temperature with 10 K increments by running 10 ps simulation at each temperature. Constraints were applied during 1.4 ns equilibration simulation where the initial force constant on the C_α_ atoms of the protein was 2 kcal/mol/Å^2^ and reduced by 0.5 kcal/mol/Å^2^ for each 0.4 ns equilibration run. CHARMM-PARAM22 force field^42^ was used for the molecular dynamics (MD) simulations. After the equilibration of the system, MD simulation was run at 310^0^ K for 10 ns. The pressure was controlled by the Langevin piston method during the simulations. The timestep was set to 2 fs and the bonded interactions, the van der Waals interactions (12 Å cutoff), long-range electrostatic interactions with particle-mesh Ewald (PME) were included for calculating the total force acting on the system. The last frame of the simulation was used as the “receptor” for the docking simulations. RMSD values were obtained using the RMSD trajectory tool of VMD. Backbone atoms (C, CA, N, and O) of each residue were used for RMSD calculation by excluding the translational motions.

### Molecular docking simulations

More than 8 million small molecules with non-identified functions were used as ligands for the docking. Molecules having the following criteria were filtered to eliminate non-relevant molecules: molecules having more than 7 H-bond donors, more than 12 H-bond acceptors, more than 600 Da molecular weight, logP > 7, more than 8 rotatable bonds, less than 3 aromatic rings^43^, and less than total of 4 rings. Openbabel, Autodock4.2, Autodock Tools4^44^ and Autodock Vina^45^ programs were utilized to prepare ligands (small molecules) for the docking. Finally, more than 1million compounds were docked to target pockets by using the Autodock Vina program. The target pocket for FAD and FBXL3 binding site was determined based on the CRY-FBXL3 crystal structure^38^. The target pocket on CRY1 was constructed via Autodock Tools. The Center of the box was located on the side chain of Phe296 amino acid residue, and the grid box size was determined as 1.9 × 10^4^ Å^3^. Another target pocket was the secondary pocket of CRY1. The Center of the box was located on the side chain of Lys11 amino acid residue, and the grid box size was determined as 2.7 × 10^4^ Å^3^.

The binding energy of molecules to CRY1 was calculated by Autodock Vina which uses a novel scoring function combining the knowledge-based and empirical approaches.

$$\Delta G_{binding} = \, \Delta G_{vdw} + \, \Delta G_{elect} + \, \Delta G_{hbond} + \, \Delta G_{desolv} + \, \Delta G_{tors}$$

Δ*G*_*vdw*_: 12–6 Lennard–Jones potential function; Δ*G*_*elect*_: Coulombic with Solmajer-dielectric function; Δ*G*_*hbond*_: 12–10 Potential with Goodford Directionality; Δ*G*_*desolv*_: Stouten Pairwise Atomic Solvation Parameters; Δ*G*_*tors*_: Number of rotatable bonds.

Autodock Tools4 or PyMol (http://pymol.sourceforge.net/) software were used to visualize the docking results and protein structure, respectively.

### MTT toxicity assay

Human osteosarcoma U2OS cell lines were used for the cytotoxicity assay. Cells were cultured and passaged at 37 °C under 5% CO_2_ in 1X medium (filtered DMEM, 10% FBS, 100 μg/ml streptomycin, and 100 μg/ml penicillin and 2 mM L-Glutamine). Cells were seeded in triplicate to clear 96-well plates with 4000 cells/well then grown for 48 h. Cells were treated with molecules at desired concentrations (final DMSO concentration 0.5%) in DMEM and incubated for 48 h. Cell viability was measured by adding tetrazolium dye 3-[4,5-dimethylthiazol-2-yl]-2,5 diphenyl tetrazolium bromide (MTT) which is converted to insoluble purple color formazan because of the mitochondrial activity. Cells were incubated with MTT reagent for 4 h and then the medium was replaced with DMSO:EtOH (50:50) mixture. Purple salt was dissolved, and the absorbance of wells was measured at 570 nm by the spectrophotometer. As a negative control, cells treated with 5% final DMSO concentration (known as toxic to cells). In each experiment 3-technical replicates were done.

### Real time bioluminescence monitoring

5 × 10^4^ U2OS *Bmal1-*d*Luc* cells per well were seeded to an opaque 96-well plate and cultured overnight as described earlier^46^. The next day cells were reset by adding dexamethasone (DXM) (0.1 µM final) for 2 h. Then medium was changed to bioluminescence recording media which contains the following in 1L: DMEM powder (sigma D-2902, 10X 1L), 0.35 gr sodium bi-carbonate (tissue culture grade, sigma S5761), 3.5gr D(+) glucose powder (tissue culture grade, sigma G7021), 10 mL 1 M HEPES buffer (Gibco 15,140–122), 2.5 mL Pen/Strep (100 ug/ml), 50 mL 5% FBS and up to 1L sterile milliQ water. Luciferin is added freshly with 0.1 mM final concentration. Molecules were added to the bioluminescence recording media at the desired concentration (0.5% DMSO final concentration). Plates were sealed with optically clear film to prevent evaporation and gas exchange thereby maintaining homeostasis of the cells. Luminescence values were recorded at 32 °C for every 30 min with 15 s integration time via Synergy H1 luminometer for a week. The experiment was repeated three times with 3-technical replicates. To obtain the period values BioDare2 (biodare2.ed.ac.uk) was used^47^. Significant analysis was performed by using the unpaired t-test with Welch’s correction.

### Establishment of CRY1-knockout U2OS cell line

*CRY1* knockout U2OS cell line was generated using the LentiCRISPRv2 system^48^. In this study, we used the LentiCRISPRv2-CRY1-T1 construct which was described previously^49^. This construct was generated using the following oligos: CRY1 Sense: 5′ CACCGCCTTCAGG GCGGGGTTGTCG 3′; CRY1 Antisense: 5′ AAACCGACAACCCCGCCCTGAAGGC 3’.

The lentivirus preparation, transduction of U2OS cells and selection of the knockout candidates with puromycin (at 0.5 mg/mL concentration) were performed as described previously^49^. *CRY1* knockout candidates were screened with immunoblotting using anti-CRY1. To show the specificity of targeting *CRY1*, we also analyzed CRY2 protein level and actin level, which was probed as the loading control. The antibodies used for this were as follow: anti-CRY1 (A302-614A, Bethyl Labs Inc. Montgomery, TX., USA), anti-CRY2 (A302-615A, Bethyl Labs), and anti-Actin (CST- 4967S, Cell Signaling Technology, Boston, MA, USA). HRP-labeled anti-rabbit antibody (Thermo Fisher Scientific, Waltham, MA, USA cat: 31460) were used at 1:5000 dilution. Chemiluminescence was developed using WesternBright Sirius HRP substrate (Advansta, San Jose, CA, USA, cat no: K-12043-D20) and images were captured using the ChemiDoc XRS + system (Bio-Rad).

### Real time bioluminescence of CRY1-knockout cells

40 × 10^4^ *Cry1*^*-/-*^ U2OS cells were seeded to 35 mm clear plates. Then, cells were transduced with *Bmal1*-d*Luc* lentiviral particles as described in Doruk et al^22^. Next cells were reset with dexamethasone (0.1 μM final) for 2 h and then media replaced with bioluminescence media described above with DMSO or molecules (final DMSO concentration 0.5%). Plates were sealed with vacuum grease and placed to luminometer LumiCycle (Actimetrics). Each plate was recorded continuously every 10 min for 70 s at 37 °C via photomultiplier tubes for a week. Raw luminescence data were analyzed using BioDare2 (biodare2.ed.ac.uk)^50^. For each molecule, the experiment was performed three times with duplicates (at least 6 plates per molecule) The unpaired t-test with Welch’s correction was used to evaluate the significance.

### Classification

PaDEL descriptors of molecules were produced using ChemDes web server^51^. The 1538 descriptors were evaluated to describe the properties of molecules; details of molecular descriptors analyzed by PaDEL in ChemDes server were given in Table 1. The molecules both in the toxicity and period change datasets belong to two groups and we can categorize these datasets using binary classification, a machine learning approach to classify objects into two groups. The toxicity molecule set is composed of toxic and nontoxic molecules whereas in the period change dataset we have group of molecules that significantly change the period and another that does not affect it. The class membership of each molecule is explained in Results and discussion section.

**Table 1:** Features of PaDEL descriptors used in this study.

Type of descriptors	Number of descriptors
E-state	568
Autocorrelation	346
Topological	266
Constitutional	120
Burden	96
Connectivity	56
Basak	42
Molecular property	15
Amino acid count	13
BCUT	6
Quantum chemical	6
Kappa	3
IP Molecular Learning	1

The number of the molecular descriptors (and so is the size of the feature space) 1538, is high relative to the number of molecules in both datasets that may cause overfitting. As such, a classifier with a good fit on the training set may produce poor results on the test dataset. To prevent overfitting, it is necessary to select the best set of molecular descriptors and eliminate the redundant features. As an initial step, the features with a single value for all the molecules are discarded since they do not provide any information for classification. For feature selection, we used Recursive Feature Elimination (RFE)^52^, which is originally proposed for selection of gene subset from patterns of gene expression data.

RFE necessitates an external estimator to weigh the features with respect to their importance. Starting from initial feature set, the estimator is trained on the current set to get the importance of each feature and the features with the least importance are discarded. The process continues the reduced sets until a feature set with a predefined size is reached.

Decision Tree (DTC)^53^, Random Forest (RFC)^54^, Extra Trees (ETC)^55^, and Gradient Boosting^56^. Classifiers were used as classification methods, all of which can also work as an estimator for RFE. DTC assigns labels to samples on leaves of a decision tree by partitioning the feature space on each node and it is superior to other methods considering its interpretability. RFC is an ensemble classification method where multiple DTCs are trained on several subsets of the dataset and prediction is made based on the outcome of individual trees. Like RFC, ETC is based on training several DTCs. The main difference is that ETC uses the full learning set instead of its subsets. In addition, to find the best split at any node ETC uses randomly selected features. As in RFC, ETC does final prediction by majority voting of the individual trees. Gradient Boosting Classifier is a boosting algorithm that converts weak learners to stronger ones. Starting from a weak learner, decision trees, it adds new trees sequentially by minimizing a loss function using a gradient descent procedure. We used Extreme Gradient Boosting^57^ (XGBC) which is an efficient, and flexible implementation of Gradient Boosting. We implemented RFE and the classification methods using the Scikit-learn package^58^ and coded in Python.

## Results and discussion

### Structure-based small molecule design

**Figure 1:**
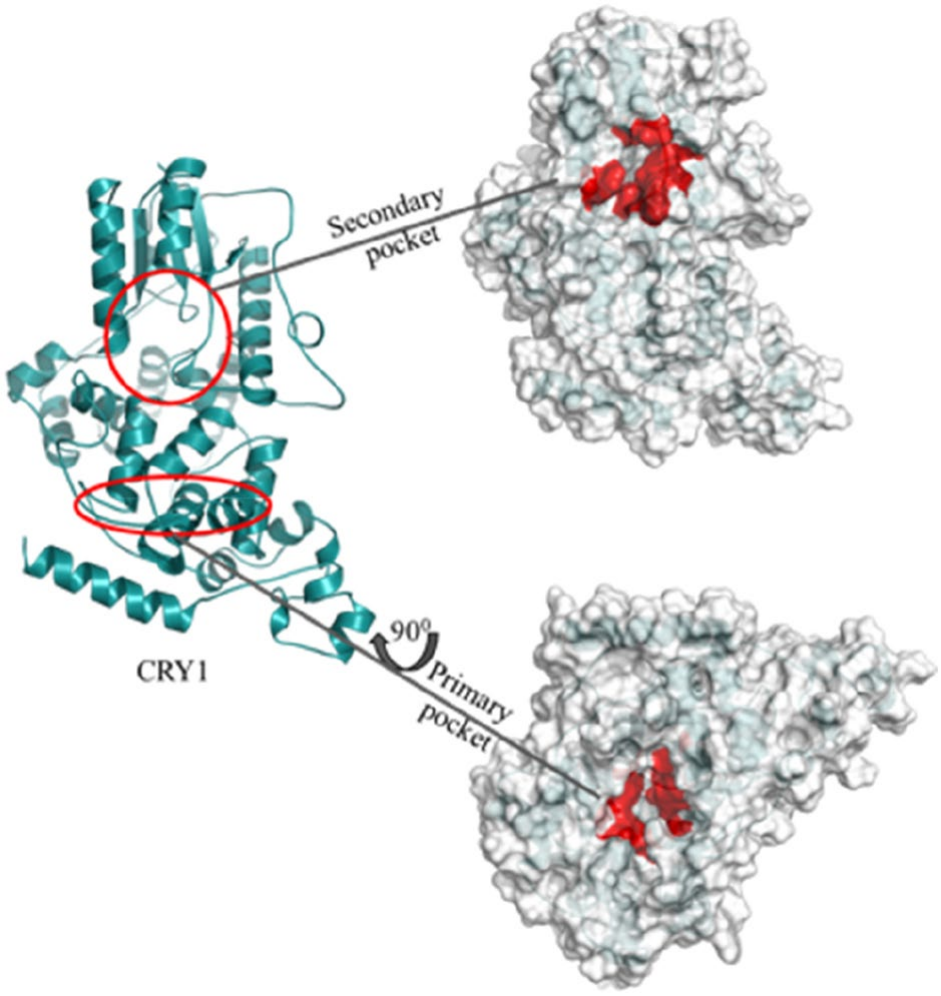
Crystal structure of Cryptochrome 1(CRY1). There are two functionally important pockets, called primary and secondary pockets. Regions in primary and secondary pockets on CRY1 are shown in red color.

CRYs are core clock proteins that participate in generating circadian rhythm by acting as strong transcriptional repressors of BMAL1/CLOCK transactivation in mammals^4,59,60^. Studies revealed that CRYs SNPs are associated with different types of diseases. For example, *CRY1* variants have been associated with depression and mood disorders^8,61,62^, elevated blood pressure and hypertension^62^. Additionally, a CRY1 variant is linked with familial delayed sleep phase disorder and attention deficit/hyperactivity disorder^18,63^. We, therefore, selected mammalian CRY1 as a target for in silico screening to find molecules that regulate the period of the circadian rhythm. The CRY1 crystal structure (ID: 4K0R) is solved^38^. Comparison of various CRYs from different organisms shows that CRYs have variable length of extended C-terminal domains that range from 30 to 300 amino acids^3,64,65^ (Fig. 1). N-terminal domain has high homology to photolyases and is called the PHR domain. The PHR domain consists of two important regions, called the FAD-binding domain (primary pocket) and an α/β domain (secondary pocket) which are shown to be important for the interaction with the FBXL3 and the CLOCK PAS B domain, respectively^66^. Therefore, the FAD-binding and secondary pockets were selected as targets which are shown to be important for regulating repressor activity of the CRYs^67,68^ (Fig. 1).

To bring CRY1 structure (PDB ID: 4K0R) near physiological conditions it was minimized and gradually heated to 310^0^ K. Then 10 ns MD simulation was run to obtain structure for the molecular docking simulations. To monitor the convergence of the simulation root mean square deviation (RMSD) of backbone atoms (C, N, C_α_) of amino acid residues were analyzed throughout the simulation (Fig. S1).

We initiated in silico screening using a commercially available small molecule library (which contains ~ 8 million molecules). Since docking pockets are large enough to accommodate relatively large molecules, we filtered the library to eliminate irrelevant molecules as described in the material-method section. Thus, nearly ~ 1 million molecules were docked to primary and secondary pockets of CRY1 by using AutodockVina. Then, molecules were ranked based on their Vina binding energies. Additionally, Pan Assay INterference compoundS (PAINS) PAINS-Remover^69^ was used to eliminate possible false-positive results. We tested 139 molecules designed for the primary packet of the CRY1 based on their availability. Similarly, 32 molecules designed for the secondary packet of the CRY1 were also tested for toxicity (Table S1).

### Toxicity of molecules

**Figure 2:**
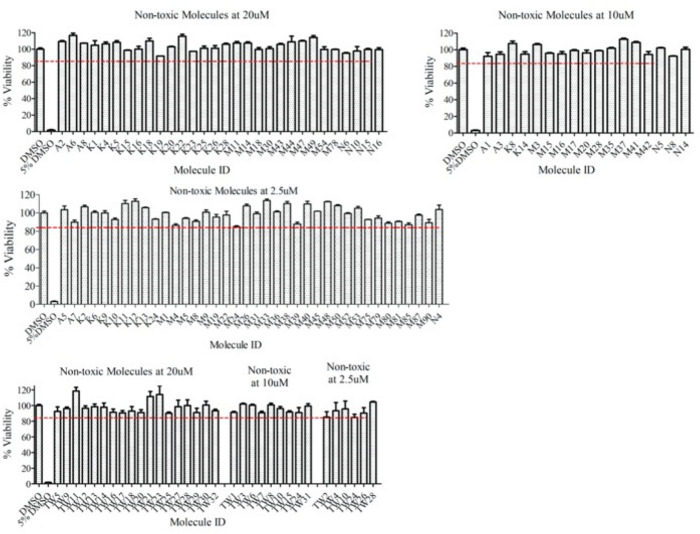
Non-toxic dosages of molecules determined by MTT toxicity assay. The cell viability was measured in cells treated with different concentrations of molecules. A dose of a molecule that allowed > 90% of cell survival evaluated as non-toxic dose. (Data represent the mean ± SEM n = 3). Cells were seeded 96-well plate and grown for 48 h. Then, cells were treated with molecules with indicated concentrations or solvent (DMSO) as control (final volume of DMSO is 0.5%). After 48 h of treatment, medium was replaced with DMEM: MTT reagent mix and incubated for 4 h. Finally, formazan salts dissolved in ethanol: DMSO mix and absorbance values of each well were measured at 570 nm using Synergy H1 (BioTek). The viability of DMSO-treated cells was normalized to 100% and the relative viability of cells treated with molecules was reported. A group of cells treated with 5% DMSO, known as toxic to cells, used as a positive control.

The toxicity studies were conducted using the human osteosarcoma (U2OS) cell line, which was also employed in the circadian bioluminescence assay. We initially tested the toxicity of the 171 compounds using an MTT (3-[4,5-dimethylthiazol-2-yl]-2,5 diphenyl tetrazolium bromide)-based assay at 20 µM and determined that 48 of them were non-toxic (Fig. 2). The remaining 123 molecules that show toxic effects at 20 µM were further evaluated at 10 µM. Results indicated that 26 molecules were not toxic at 10 µM. Finally, the other 97 molecules were tested at 2.5 µM and found that 41 molecules were non-toxic at this concentration (Fig. 2). The rest of 56 molecules with relative cell viability < 85% at 2.5 µM were labeled as toxic and, therefore, eliminated from further characterization. As a control, cells treated with 5% DMSO known to be toxic. In summary, of 171 tested molecules, 56 were toxic to U2OS cell lines whereas the 115 molecules were evaluated as non-toxic molecules at different concentrations. Structures of all molecules were provided in the supplementary data (Fig. S2).

### Classification of molecules based on toxicity

The toxicity data set is composed of 171 molecules with 1538 molecular descriptors. 334 features repeating the same value for all molecules were discarded. The remaining 1203 features were utilized to obtain the best feature set by Recursive Feature Elimination (RFE). Since RFE method^52^ and so the selected feature set is dependent on the estimator used, Decision Tree Classifier (DTC)^53^, Random Forest Classifier (RFC)^54^, Extra Trees Classifier (ETC)^55^, and Gradient Boosting (XGBC)^56^ were tested as the external estimators. To search for the promising regions in the space of the selected features, we generated feature sets with cardinality ranging from 2 to 20 in increments of 1 for each of these classifiers. To evaluate the potency of the selected features and compare the classifiers based on their prediction accuracy, 10-Fold cross-validation (CV) were run on all the generated sets and replicated 100 times.

The toxic and non-toxic groups of molecules are not evenly distributed. The 33% of all molecules are toxic and the rest, 67%, is non-toxic. To cope with the unbalanced groups, we used weights associated with each class which are inversely proportional to the class sizes. The weights corresponding to toxic and non-toxic molecules, 1.53 and 0.74 are calculated by *w*_*T*_ = *n*/(2*n_*T*_), *w*_*NT*_ = *n*/(2*n_*NT*_), respectively, where *n* is the total number of molecules, *n*_*T*_ and *n*_*NT*_ are the number of toxic and non-toxic molecules in the dataset.

To optimize the performance in discriminating between toxic and non-toxic molecules, we tuned the hyperparameters of each classifier. We did a grid search within the space of all combinations of a selected set of values of parameters and optimized over 10-Fold CV. The parameters of DTC and their corresponding values evaluated in grid search are, max_depth (The maximum depth of the tree): [1, 2, 3, … ,10, None], min_samples_splits (the minimum number of samples required to split an internal node): [2, 3, … ,10], min_samples_leafs (The minimum number of samples required to be at a leaf node): [1, 2, 3, … ,10], and max_features (the number of features to consider when looking for the best split): [1, 2, 3, … , num_features]. RFE and ETC parameters and their alternative values are max_depth: [1, 2, 3, … ,6, None], min_samples_splits: [2–5], min_samples_leafs: [1–5], max_features: [1, 2, 3, … , sqrt(num_features)], and n_estimators (The number of trees in the forest): [100, 200]. Note that the first four parameters of RFC and ETC are common with DTC. However, we use a smaller space of values due to the computational complexity of RFC and ETC. The set of parameters of XGBC and their set of levels to optimize are learning_rate (step size shrinkage used in update to prevents overfitting): [0.01, 0.1], max_depth: [3, 5, 7, 10], min_child_weight (minimum sum of instance weight needed in a child): [1, 3,5], subsample (subsample ratio of the training instances): [0.5, 0.7], colsample_bytree (subsample ratio of columns when constructing each tree): [0.5, 0.7], and n_estimators: [100, 200]. We used gbtree as the booster of XGBC which employs tree-based models.

To search for the promising regions in the space of the selected features, we generated feature sets with cardinality ranging from 2 to 20 in increments of 1 for each of the classifiers. To evaluate the potency of the selected features and compare the classifiers based on their prediction accuracy, 10-Fold cross-validation (CV) were run on all the generated sets and replicated 100 times. The grid search for parameter tuning is made for each feature set independently and CV repetitions are implemented based on tuned hyperparameters.

The average accuracies of classifications with 100 replications on generated feature sets are given in Table 2. The rows represent the number of features selected and the mean accuracies for the generated feature sets. The classifiers used for 10-Fold CV are placed in columns. The maximum average accuracies for each of the classifiers are marked in bold numbers. Our analyses show that DTC attained the highest mean accuracy of 78.77% for 19 feature set and is by far superior to the other classifiers studied in terms of prediction power. RFC follows DTC with the highest mean accuracy 72.99% for a set having 9 features, while ETC and XGBC are inferior, resulting in 71.36% and 71.17% maximum accuracies with 20 and 17 features, respectively. The maximum and standard deviation of 100 CV accuracies for each feature set and classifier pair are presented in Tables S2 and S3, respectively. In line with the mean accuracy comparison, DTC attained the best with 84.80% maximum accuracy on a set with 19 features. RFC and XGBC reached the highest score of 77.78% while ETC stayed at 76.02% level. The standard deviation of DTC values is greater than 2 except for one feature set and is higher compared to the other classifiers (Table S3). However, this high variation in DTC results is compensated by higher mean accuracies. The lower variation in RFC, ETC and XGBC results, mostly less than 2, does not pose an advantage due to their lower mean accuracies.

**Table 2:** Toxicity dataset, mean accuracy of 10-Fold CV with 100 replications for feature sets with cardinality ranging from 2 to 20.

Features	10 Fold CV—accuracy (%)			
	DTC	RFC	ETC	XGBC
2	60.22	68.63	70.32	65.06
3	59.20	68.68	70.40	67.64
4	60.39	71.97	68.82	68.10
5	71.93	69.47	69.88	68.88
6	69.87	71.18	67.99	68.67
7	71.73	72.70	68.12	68.20
8	72.81	72.78	69.16	67.37
9	75.87	**72.99**	68.16	68.32
10	75.65	72.68	70.21	67.92
11	76.75	71.71	68.60	70.18
12	75.33	71.43	68.83	70.16
13	76.46	72.36	68.57	68.87
14	78.49	72.54	68.23	68.63
15	77.22	70.76	69.67	69.08
16	77.21	72.41	68.73	71.16
17	75.83	72.81	68.77	**71.17**
18	78.75	72.37	69.49	71.05
19	**78.77**	70.03	70.32	70.11
20	78.02	70.73	**71.36**	70.73

DTC, RFC, ETC, and XGBC trained and tested on feature sets with cardinality between 2 and 20.

The feature set with cardinality 14 results in a mean accuracy of 78.49% by DTC and it is very close to the highest score of 78.77% for 19 features. We concluded that the additional 5 features do not provide significant improvement in the prediction power of DTC and we continued our study with 14 features. Tuning Hyperparameters of DTC by grid search for 14 features resulted in the optimized values: max_depth = None, max_features = 13, min_samples_leaf' = 1, min_samples_split = 5.

The RFE method iteratively prunes the least important features to get the set with preferred cardinality. However, the generated set is not guaranteed to be optimal. To determine the most essential features, we iteratively pruned the features in a similar approach with RFE in the selected 14 features. At each iteration, we did 100 CV repetitions on the reduced sets obtained by dropping every feature one at a time. The feature that provides the highest mean accuracy among the reduced sets was pruned. Our analysis for the reduced sets together with the pruned molecular descriptor showed that removing the descriptor ATSC8v to get 13 features increased the mean accuracy from 78.49 to 79.63% (Table 3). Further reduction in the size of the feature set decreased the mean accuracies. This is probably due to excluding the informative descriptors. We concluded that 13 features are the best descriptive set among 1203 descriptors with DTC to classify the toxicity data. Note that since there is no max_depth limit for 14 features DTC parameters, at each pruning step we additionally tuned the max_depth parameter to get the best max_depth level of 10.

**Table 3:** Toxicity Dataset, maximum, mean, and standard deviation of 10-Fold CV accuracies with 100 repetitions.

Features	Removed	Max	Mean	Std. Dev
14	-	83.04	78.49	2.36
13	ATSC8v	84.21	79.63	2.01
12	VE3_Dt	84.21	79.41	2.50
11	SpMin1_Bhs	82.46	78.90	1.77
10	SpMax5_Bhv	83.63	78.95	1.86
9	GATS8e	83.04	77.82	2.17

DTC applied to reduced feature sets obtained by removal of a single feature at a time.

**Figure 3:**
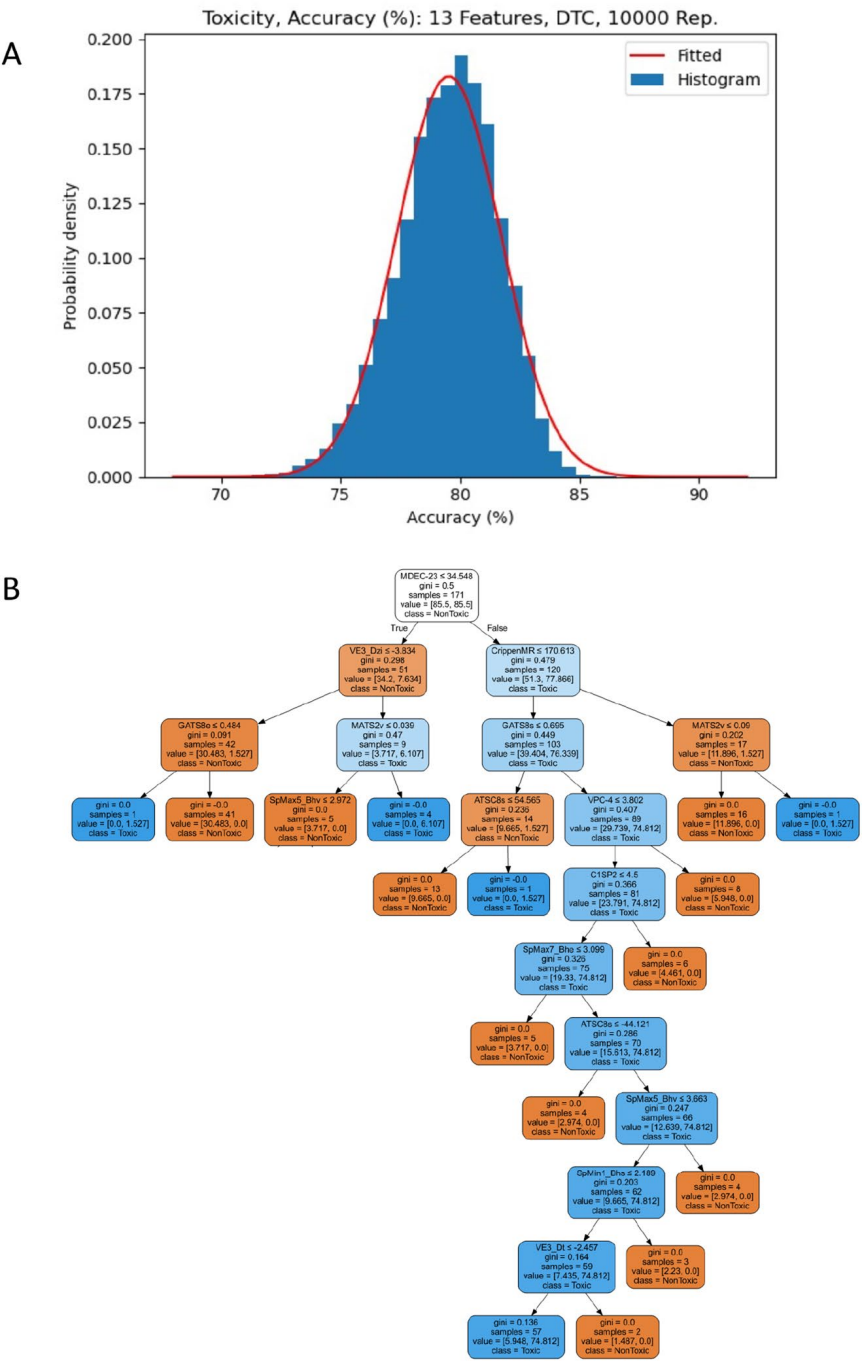
(**A**) Toxicity Dataset, histogram and fitted normal probability density function of the accuracies for 10,000 replications of DTC (Max. Depth, 10) applied to final 13 molecular descriptors. (**B**) Toxicity Dataset, DTC plot^70^, which is generated by graphviz.org, with maximum depth 10 trained on final 13 features.

The selected 13 molecular descriptors are: “MDEC-23, MATS2v, ATSC8s, VE3_Dt, CrippenMR, SpMax7_Bhe, SpMin1_Bhs, C1SP2, GATS8e, GATS8s, SpMax5_Bhv, VE3_Dzi, VPC-4.” (Table 4). Finally, 10,000 CV repetitions were run to get the maximum and mean accuracies and the standard deviation of accuracies i.e. 86.55, 79.53, 2.18, respectively. The accuracies displayed an approximately normal distribution for the histogram of accuracies and probability density function of the fitted normal distribution (Fig. 3A). The plot of DTC with *d*_*max*_ 10 trained on 13 features showed that among 16 leaf nodes, 11 (orange color) conclude that a new molecule is nontoxic, and the rest 5 (blue color) results in the decision that it is toxic (Fig. 3B).

**Table 4:** Name, type, and description of selected 13 features determining the toxicity of a molecule.

Descriptor name	Type	Description
MDEC-23	MDEDescriptor	Molecular distance edge between all secondary and tertiary carbons
MATS2v	Moran Autocorrelation Descriptor	Moran autocorrelation—lag 2/weighted by van der Waals volumes
ATSC8s	Centered Broto-Moreau Autocorrelation Descriptor	Centered Broto-Moreau autocorrelation—lag 8/weighted by I-state
VE3_Dt	Detour Matrix Descriptor	Logarithmic coefficient sum of the last eigenvector from detour matrix
CrippenMR	Crippen Descriptor	Crippen's molar refractivity
SpMax7_Bhe	Burden Modified Eigenvalues Descriptor	Largest absolute eigenvalue of Burden modified matrix—n 7/weighted by relative Sanderson electronegativities
SpMin1_Bhs	Burden Modified Eigenvalues Descriptor	Smallest absolute eigenvalue of Burden modified matrix—n 1/weighted by relative I-state
C1SP2	Carbon Types Descriptor	Doubly bound carbon bound to one other carbon
GATS8e	Geary Autocorrelation Descriptor	Geary autocorrelation—lag 8/weighted by Sanderson electronegativities
SpMax5_Bhv	Burden Modified Eigenvalues Descriptor	Largest absolute eigenvalue of Burden modified matrix—n 5/weighted by relative van der Waals volumes
VE3_Dzi	Barysz Matrix Descriptor	Logarithmic coefficient sum of the last eigenvector from Barysz matrix/weighted by first ionization potential
VPC-4	ChiPath Cluster Descriptor	Valence path cluster, order 4

### Circadian bioluminescence assay

U2OS cell is a commonly used cell line in the circadian rhythm field due to its robust rhythm^22,25,46,71^. Any agents such as small interfering RNA (siRNA) and chemicals or gene knockout (KO) affecting the stability or activity of clock proteins change the parameters (period, amplitude, and phase) of the circadian rhythm^25,72^. We analyzed the effect of non-toxic molecules on the period length of circadian rhythm in U2OS cells stably expressing destabilized firefly Luciferase (d*luc*) under the control of the *Bmal1* promotor (U2OS *Bmal1-*d*luc*).

**Figure 4:**
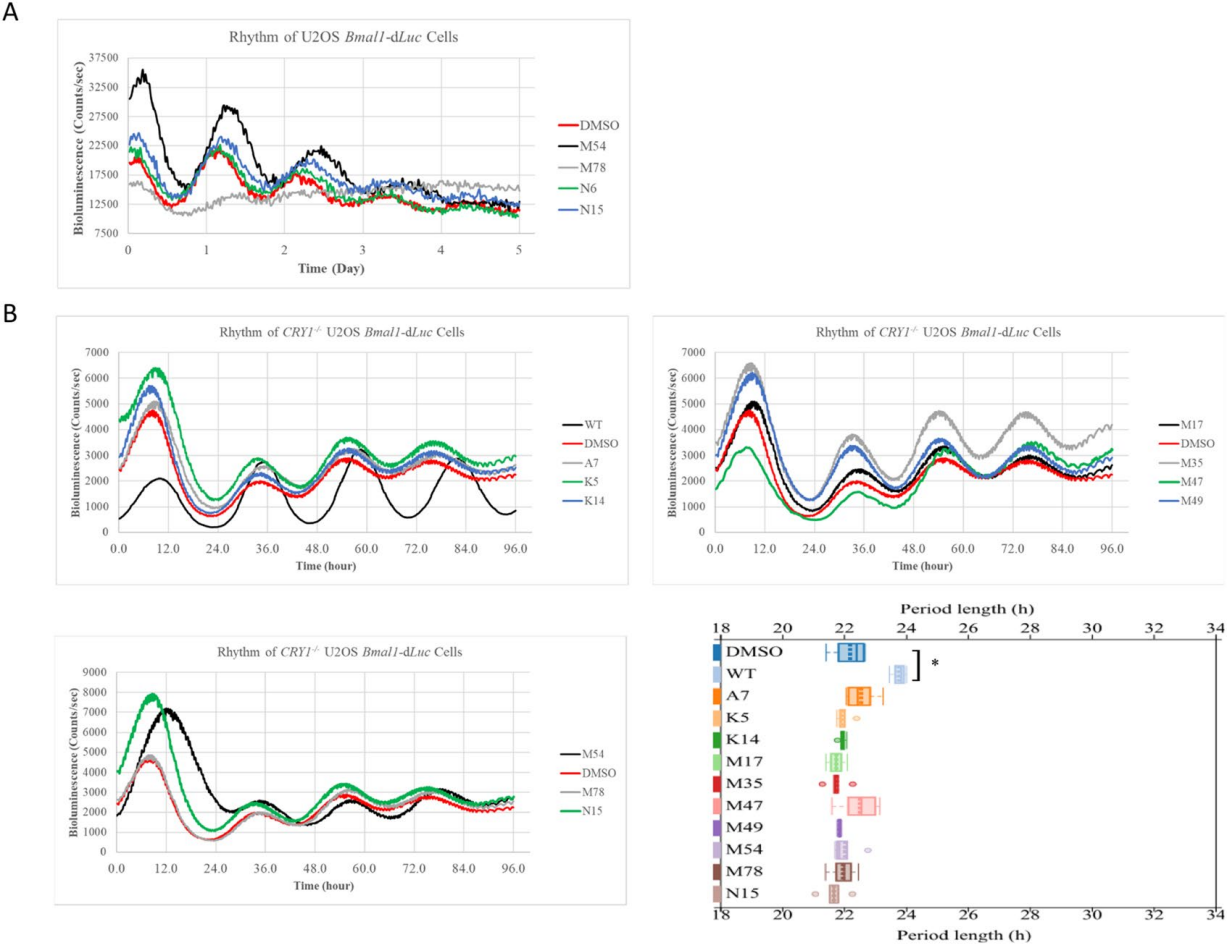
The effect of the molecules on circadian rhythm. (**A**) 5 × 10^4^ U2OS *Bmal1*-d*Luc* cells were seeded to an opaque 96-well plate. Next day cells were synchronized by dexamethasone for 2 h. Then the medium was replaced with luminescence recording medium having molecules or DMSO. Bioluminescence readings were recorded for a week using Synergy H1 (BioTek). Period data was calculated using Biodare2 web-server (biodare2.ed.ac.uk) (all results were given in Fig. S3). To determine molecules that are changing the period of the rhythm significantly, the period length of molecule-treated cells was compared to that of DMSO control using unpaired t-test with Welch’s correction (****p < 0.0001 ***p = 0.001 **p < 0.01, *p < 0.05, n = 3). Each biological replicate was the average of the 3 technical replicates. (**B**) 1 × 10^5^ *CRY1*^*-/-*^U2OS transduced with *Bmal1-*d*Luc* reporter. WT represents U2OS *Bmal1*-d*Luc.* Bioluminescence readings were recorded for a week using LumiCycle (Actimetrics, USA). Period data was calculated using Biodare2 web-server (biodare2.ed.ac.uk). Unpaired t-test with Welch’s correction was used for significant analysis (*p < 0.05, n = 3). Each biological replicate was the average of the two technical replicates.

Since the primary and secondary pockets of CRY1 are critical to interact with different proteins e.g. CLOCK and FBXL3, respectively, molecules designed for these two pockets might have differential impacts on the circadian rhythm. Thus, we focused only on the effect of 85 non-toxic molecules designed for the primary pocket of CRY1. U2OS *Bmal1*-d*Luc* cells treated with these molecules and their effect on circadian period length was analyzed by BioDare2 (biodare2.ed.ac.uk)^50^. Analysis revealed that 21 molecules significantly lengthen the period of circadian rhythm (Fig. S3). One molecule, N8, shortened the period and was excluded from further classification studies. The representative figure for period lengthener molecules is shown in Fig. 4A. Circadian rhythm results of all period lengtheners were given in Fig. S3. All period values were provided in Table S4. To verify the CRY1 dependency of molecules, we generated U2OS *CRY1*^*-/-*^* Bmal1*-d*Luc* cells by utilizing CRISPR/Cas9 technology (Fig. S4). Knocking out the *CRY1* in this cell line resulted in a shorter period (indicated with red line) compared to wild-type controls (indicated with black line) as in agreement with previously published data^73^ (Fig. 4B). Notably, when U2OS *CRY1*^*-/-*^*Bmal1*-d*Luc* cells were treated with potent molecules A7, K5, K14, M17, M35, M47, M49, M54, M78, and N15 no change was observed in the period length of the circadian rhythm (Fig. 4B). We, then, performed a classification study to determine the molecular characteristics leading to period change.

**Figure 5:**
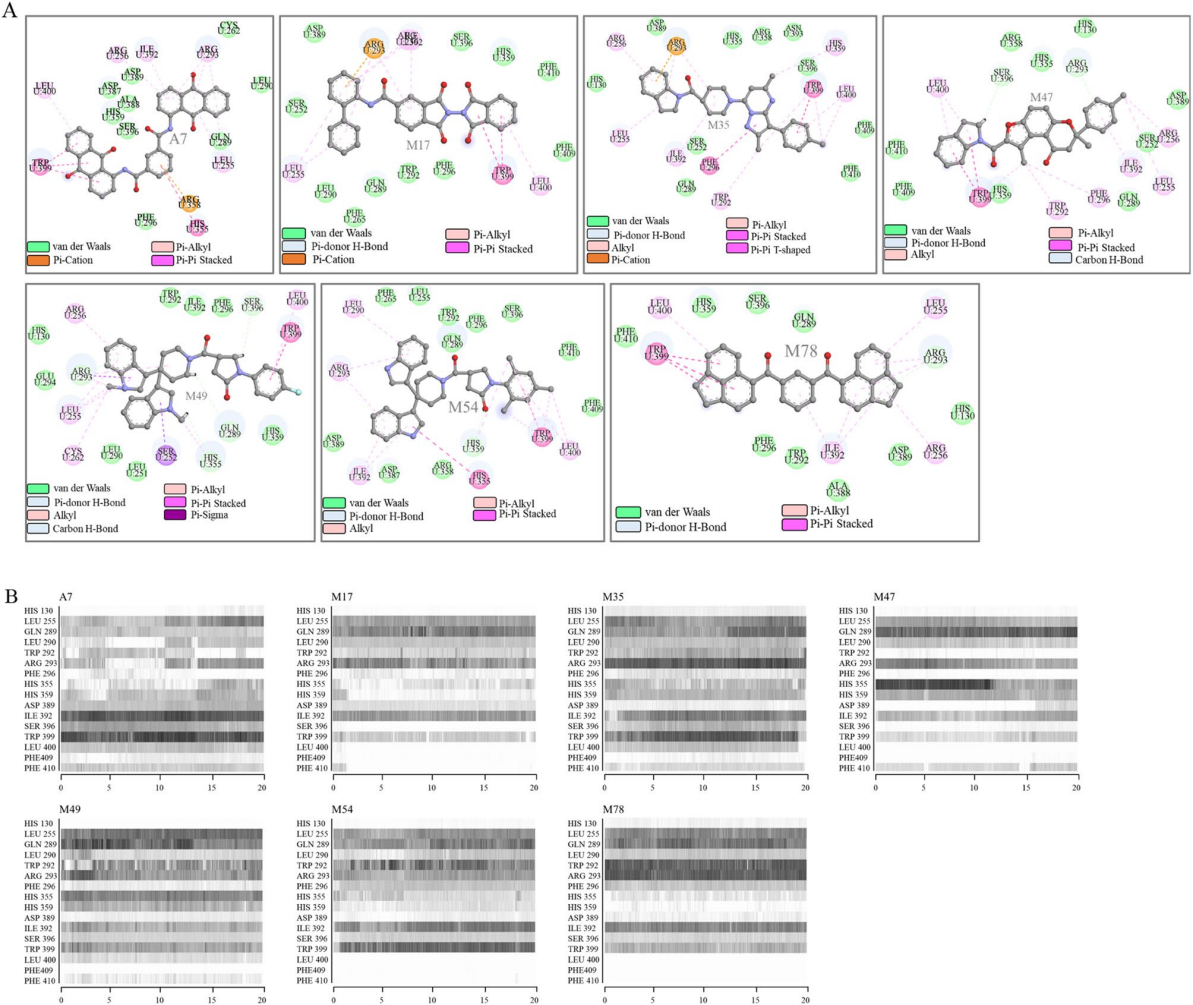
Binding mode of molecules on CRY1 and analysis of molecular dynamic simulations of CRY1-molecule complexes. (**A**) Docked conformation of molecules was analyzed and 2D interaction map was generated using Discovery Studio Visualizer. Type of interactions was given in color-coded format. (**B**) Interaction maps of molecules with CRY1 through 20 ns MD simulation were generated using VMD timeline applet. It has been evaluated as interaction if molecule and amino acid residues get 5 Å or closer and shown with a black bar.

Next, we analyzed the stability of the interaction between molecules and CRY1 using the MD simulations. The seven of the most potent (A7, M17, M35, M47, M49, M54, and M78) molecules in complex with CRY1 were simulated. Parameters for molecules were generated using CHARMM-GUI server. The CRY1-molecule complexes, obtained from docking analysis, were simulated for 20 ns. The initial docking position of molecules and nearby amino acid residues on CRY1 were shown in (Fig. 5A). We identified the nature of interactions between CRY1 and molecules as followings. Ring structures in molecules generated pi-type (Pi-cation, Pi-alkyl, Pi-Pi stacked or T-shaped) interactions with at least one of either Arg-293, His-355 and Trp-399 of CRY1. In addition, all molecules interacted with Leu-255 and Ile-392 of the CRY1 through van der Waals forces. To evaluate the persistence of these interactions we generated a contact map of molecules with nearby amino acids through 20 ns MD simulations. RMSD values of backbone atoms of proteins showed that simulations reached the equilibrium (Fig. S5). Contact maps showed that these interactions were maintained during the simulations (Fig. 5B). Visual inspection showed that molecules did not cause any conformational changes and their interactions were maintained throughout the simulation. A list of highly interacting amino acids with each molecule was given in Table 5. In addition, amino acid residues, which formed hydrogen bonds, were determined from the initial docking position of molecules (Table 5) and the persistence of these interactions was confirmed from interaction maps (Fig. 5B).

**Table 5:** Highly interacting and hydrogen bond generating amino acid residues of CRY1 with molecules.

Molecules	Interacting amino acids	Hydrogen-bond forming amino acids
A7	Leu-255, Gln-289, Asp-389, Ile-392, Ser-396, Trp-399	Arg-293, His-359
M17	Leu-255, Gln-289, Leu290, Trp292, Arg-293, Ile-392	Arg-293
M35	Leu-255, Gln-289, Trp-292, Arg-293, Ile-392, Trp-399	Arg-293, His-359
M47	Leu-255, Gln-289, Leu-290, Arg-293, His-355, His-359	Arg-293, His-359
M49	Leu-255, Gln-289, Arg-293, His-355, Ile-392, Trp-399	Arg-293, Gln-289, His-355
M54	Leu-255, Gln-289, Trp-290, Arg-293, Ile-392, Trp-399	His-359, Ser-395
M78	Leu-255, Gln-289, Trp-292, Arg-293, Ile-392, Trp-399	Ser-396

### Classification of period lengthening molecules

Factors determining the period length in the circadian rhythm are quite complex. For example, the deletion of analogous *Cry1* and *Cry2* genes in mice causes short and long period phenotypes, respectively^74^. CRY binding small molecules discovered by high-throughput screening were reported to stabilize the CRY1. Interestingly, these molecules caused differential circadian phenotype in treated cells e.g. shorter or longer period length^25,27^. Thus, we focused only on the period lengthening molecules. Previously reported 6 molecules binding to CRY1 and lengthened the period of circadian rhythm (KL001, GO058, GO061, GO152, GO214, GO216)^25,27^ were included in the classification analysis, GO203 which does not change the rhythm included as no-changer. We started the classification with 90 molecules of which 27 are period lengthening, 63 are no-changers. 1538 molecular descriptors were generated for all molecules. However, 360 of them have the same value for all molecules and were discarded. The remaining 1177 features were used to train the dataset.

We followed a similar approach with the toxicity dataset for the classification of the set of period-lengthening molecules. The period dataset is also unbalanced as in toxicity since 30% of the molecules are period changers and the rest 70% are no-changer molecules. To deal with the possible bias, we set the weights of the period-lengthening and no-changer molecules as 1.67 and 0.71, respectively. We generated feature sets with cardinalities between 2 and 20 from the period dataset by RFE using DTC, RFC, ETC and XGBC as external estimators. Next, we tuned the parameters of the given classifiers on each of the feature sets and did 100 CV with the optimized parameters.

Mean accuracy levels for each feature set and classifier pair are presented in Table 6. All of the classifiers achieved mean accuracies greater than 80% for multiple numbers of feature sets and the highest mean accuracies for each classifier are marked in bold. RFC and XGBC are the best of all with highest mean accuracies, 83.62% for 16 features and 83.69% for 15 features. Since XGBC provided slightly higher mean accuracy than RFC with 1 less feature, we selected XGBC as the most promising classifier for the period dataset.

**Table 6:** Period lengthening dataset, mean accuracy of 10-Fold CV with 100 repetitions.

Features	10 Fold CV—accuracy (%)			
	DTC	RFC	ETC	XGBC
2	74.63	75.78	69.19	63.17
3	70.00	74.48	76.78	70.42
4	70.30	82.17	78.24	71.74
5	79.07	78.22	78.29	75.31
6	80.92	79.57	79.68	73.66
7	79.87	80.04	79.82	74.92
8	**82.18**	79.14	81.77	75.37
9	82.10	82.72	**81.91**	78.88
10	80.82	81.30	78.11	80.50
11	81.93	80.83	80.24	80.43
12	81.23	81.13	79.89	81.63
13	82.13	80.93	77.34	83.28
14	78.52	81.02	77.67	82.69
15	80.66	81.16	75.08	**83.69**
16	78.70	**83.62**	77.49	82.59
17	75.21	82.20	79.61	80.94
18	78.32	82.74	81.60	81.76
19	76.88	79.60	78.34	82.97
20	77.41	79.62	81.16	82.53

DTC, RFC, ETC, and XGBC trained and tested on feature sets with cardinality between 2 and 20.

The maximum and standard deviations of 100 CV accuracies for each feature set and classifier pair are given in Tables S5 and S6 respectively. Among all, XGBC provided the maximum accuracy of 90% again with the 15 features. XGBC Parameters tuned for 15 features are such that, colsample_bytree = 0.5, learning_rate = 0.1, max_depth = 3, min_child_weight = 1, n_estimators = 100, and subsample = 0.7.

As performed in the toxicity dataset, we iteratively pruned the features in the selected 15 features to eliminate the redundant ones, this time using XGBC with the tuned hyperparameters. In Table 7 maximum, mean and standard deviation of accuracies of 100 CV applied on reduced feature sets are presented. Reducing the set from 15 to 10 features increased the mean accuracy from 83.69 to 86.94%. Dropping further features reduced the mean accuracy since all the features in 10 features are informative.

**Table 7:** Period lengthening dataset, maximum, mean, and standard deviation of 10-Fold CV accuracies with 100 repetitions.

Features	Removed	Max	Mean	Std. Dev
15	–	90.00	83.69	2.41
14	ATS3m	90.00	85.11	2.11
13	AATSC4m	90.00	86.11	2.07
12	MATS5m	91.11	86.17	2.52
11	minHBint2	91.11	86.48	2.27
10	AATS4p	92.22	86.94	2.18
9	minsCH3	91.11	86.82	2.21
8	ATSC4p	90.00	85.47	2.16
7	MLFER_S	88.89	84.74	2.49

XGBC applied to reduced feature sets obtained by removal of a single feature at a time.

**Figure 6:**
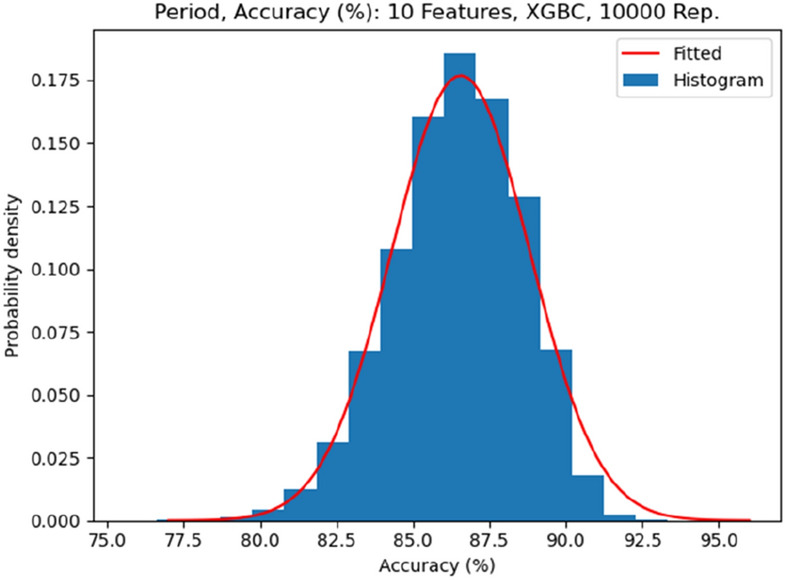
Period Change Dataset, histogram, and fitted normal probability density function of the accuracies for 10,000 replications of XGBC applied to final 10 molecular descriptors.

We concluded that XGBC with the tuned parameters coupled with the reduced set with 10 features is the best classifier for the period changer dataset. We did a final 10,000 repetition of 10-Fold CV with XGBC on 10 features to get the maximum and mean accuracies of 93.33%, and 86.56% respectively. The histogram and fitted normal probability density function of the accuracies for 10,000 replications are presented in Fig. 6. The selected final 10 features includes: “ATSC8c, MATS1e, minsCH3, MATS4e, MATS4s, ATSC7i, SpMin4_Bhp, MLFER_S, ATSC4p, SpMax2_Bhm” (Table 8).

**Table 8:** Name, type, and description of selected 10 features determining the period changing information of a molecule.

Descriptor name	Type	Description
ATSC8c	Centered Broto-Moreau Autocorrelation Descriptor	Centered Broto-Moreau autocorrelation—lag 8/weighted by charges
MATS1e	Moran Autocorrelation Descriptor	Moran autocorrelation—lag 1/weighted by Sanderson electronegativities
minsCH3	Electrotopological State Atom Type Descriptor	Minimum atom-type E-State: -CH3
MATS4e	Moran Autocorrelation Descriptor	Moran autocorrelation—lag 4/weighted by Sanderson electronegativities
MATS4s	Moran Autocorrelation Descriptor	Moran autocorrelation—lag 4/weighted by I-state
ATSC7i	Centered Broto-Moreau Autocorrelation Descriptor	Centered Broto-Moreau autocorrelation—lag 7/weighted by first ionization potential
SpMin4_Bhp	Burden Modified Eigenvalues Descriptor	Smallest absolute eigenvalue of Burden modified matrix—n 4/weighted by relative polarizabilities
MLFER_S	MLFER Descriptor	Combined dipolarity/polarizability
ATSC4p	Centered Broto-Moreau Autocorrelation Descriptor	Centered Broto-Moreau autocorrelation—lag 4/weighted by polarizabilities
SpMax2_Bhm	Burden Modified Eigenvalues Descriptor	Largest absolute eigenvalue of Burden modified matrix—n 2/weighted by relative mass

## Conclusions

Drug discovery is a very expensive and time-consuming process posing several daunting challenges. Compared to the classical high-throughput approach to computer-assisted drug discovery, employing virtual screening (VS) is a promising approach to reduce the cost of the initial drug discovery. VS allows identifying hit compounds from large databases of drug-like molecules much faster and cheaper than traditional approaches. VS utilizes comprehensive evaluation of ADMET parameters by pharmacophore modeling^75^ and quantitative structure–activity relationship (QSAR) analysis^76^. In addition to these, toxicity prediction is becoming a more significant part of current computer-assisted drug development, especially when libraries contain tens of millions of untested compounds. As a result, quick and inexpensive computational algorithms are frequently used to eliminate potentially toxic compounds and reduce the number of experimental tests required. Here we identified small molecules that bind functionally important regions of a core clock protein CRY1. First, of tested 171 molecules, 115 molecules are nontoxic while 56 molecules are toxic. Then we performed machine learning methods to classify toxic and nontoxic molecules. DTC identified 13 features that can predict the toxicity with accuracy of about 80%. Second, 21 molecules were identified as period lengthener among 85 molecules. Furthermore, machine learning approach using XGBC determined 10 molecular descriptors that can predict period lengthener molecules about 87% accuracy. These descriptors can be implemented in future VS studies on CRY1 to predict the toxicity and period lengthener effect of molecules from libraries containing several hundred million compounds.

## Supplementary Information

Supplementary Information 1.

Supplementary Information 2.

Supplementary Information 3.

Supplementary Information 4.

Supplementary Information 5.

Supplementary Information 6.

Supplementary Information 7.
