# Supplementary material for: Structure-based design and classifications of small molecules regulating the circadian rhythm period
Source: Sci Rep. 2021 Sep 16;11:18510. doi: 10.1038/s41598-021-97962-5 (PMC8445970; doi:10.1038/s41598-021-97962-5)
Supplement: Supplementary file 1 — Supplementary Information 1. [file 41598_2021_97962_MOESM1_ESM.docx]

**Supplementary Data**

**Structure-based design and classifications of small molecules regulating the circadian rhythm period**

Seref Gul^1^, Fatih Rahim^2^, Safak Isın^3^, Fatma Yılmaz^4^, Nuri Ozturk^4^, Metin Turkay^2*^, and Ibrahim Halil Kavakli^1,3*^

*Koc University Departments of ^1^Chemical and Biological Engineering, ^2^Industrial Engineering,* ^3^*Molecular Biology and Genetics, Rumelifeneri Yolu, Sariyer, Istabul, Turkey*

*^4^Department of Molecular Biology and Genetics, Gebze Technical University, Gebze, 41400, Kocaeli, Turkey*


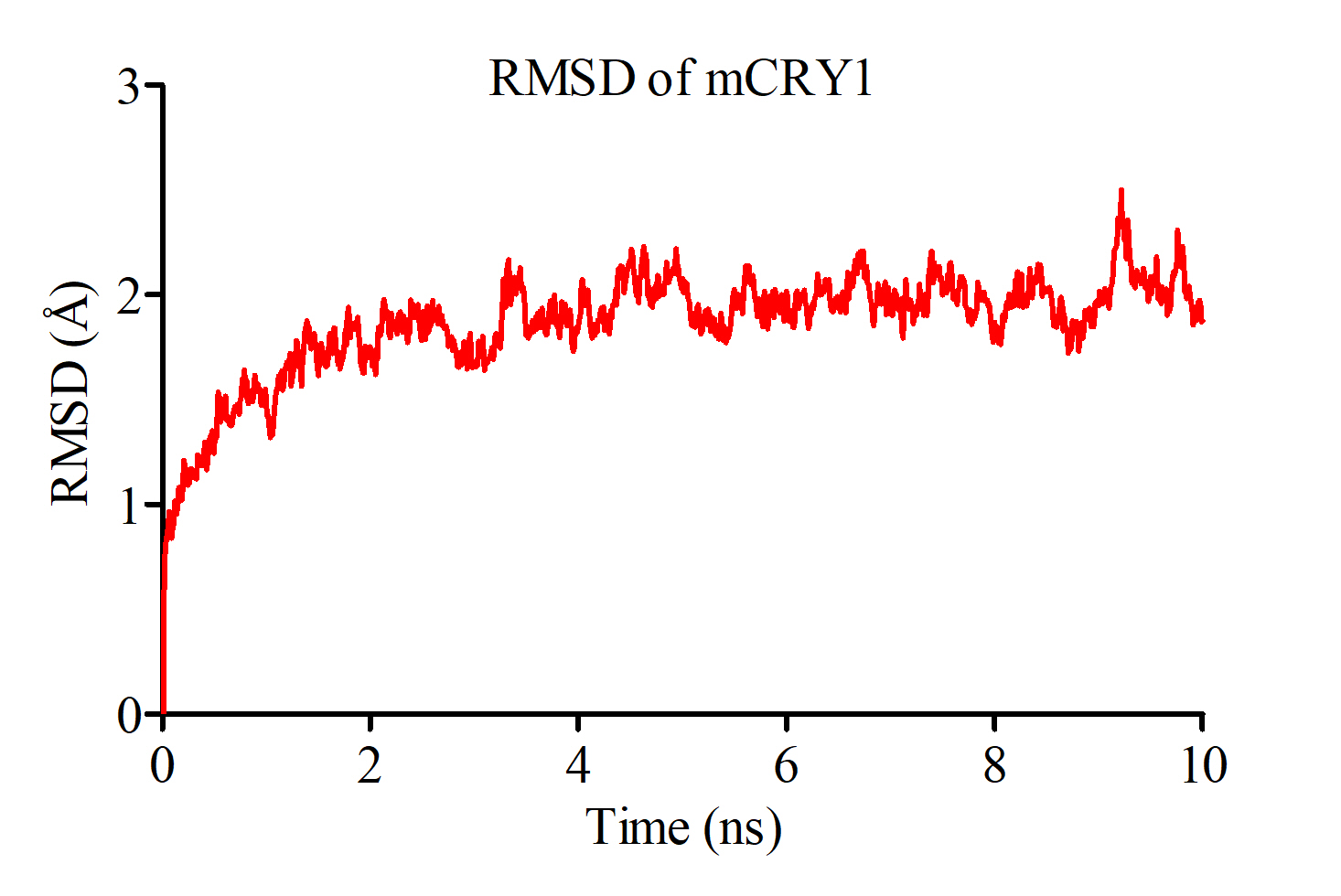


**Figure S1:** RMSD of CRY1 simulation (10ns). RMSD values of backbone atoms (N-Cα-C) for each amino acid residues were calculated.

**Figure S2**: The structures of the molecules.

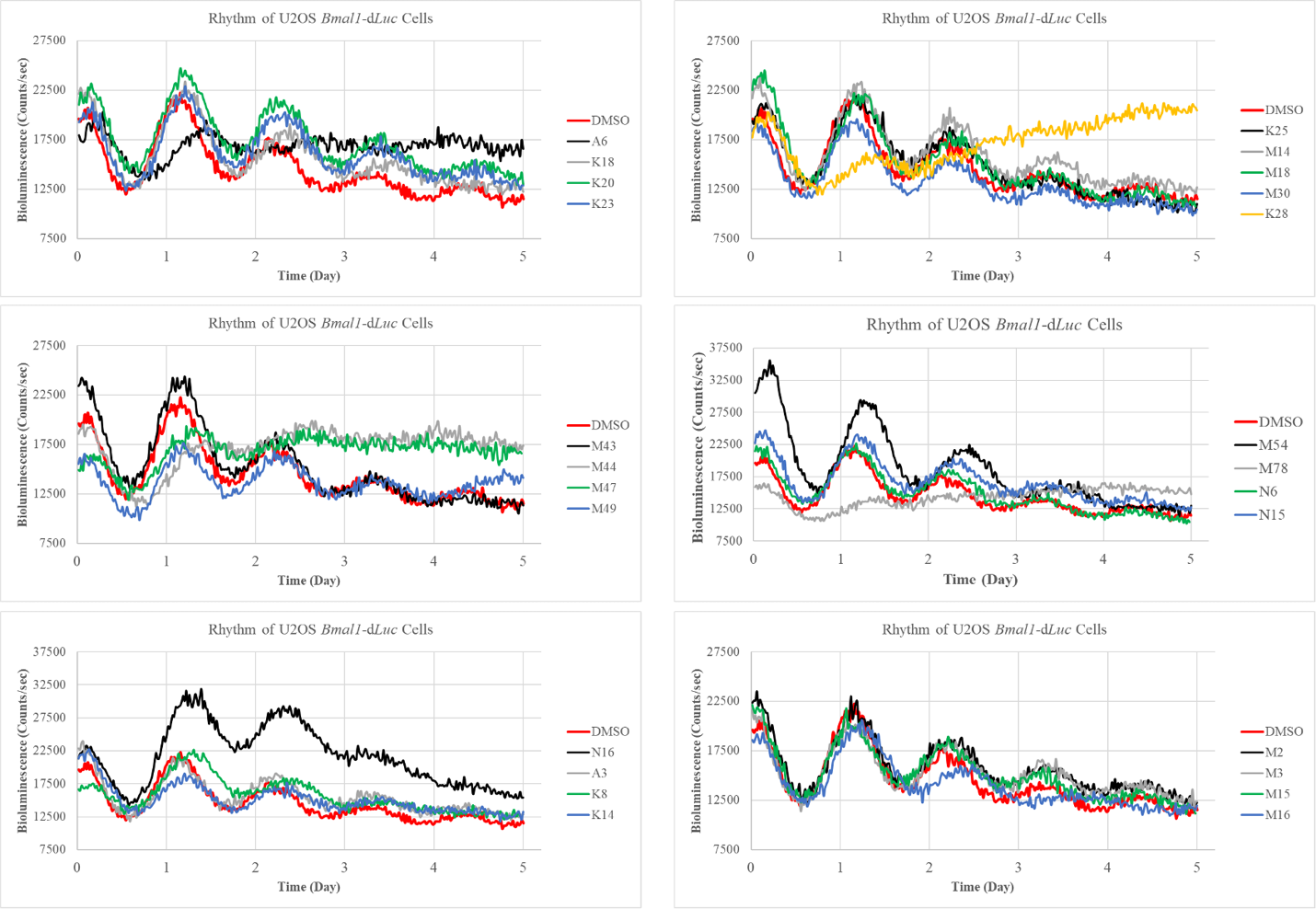


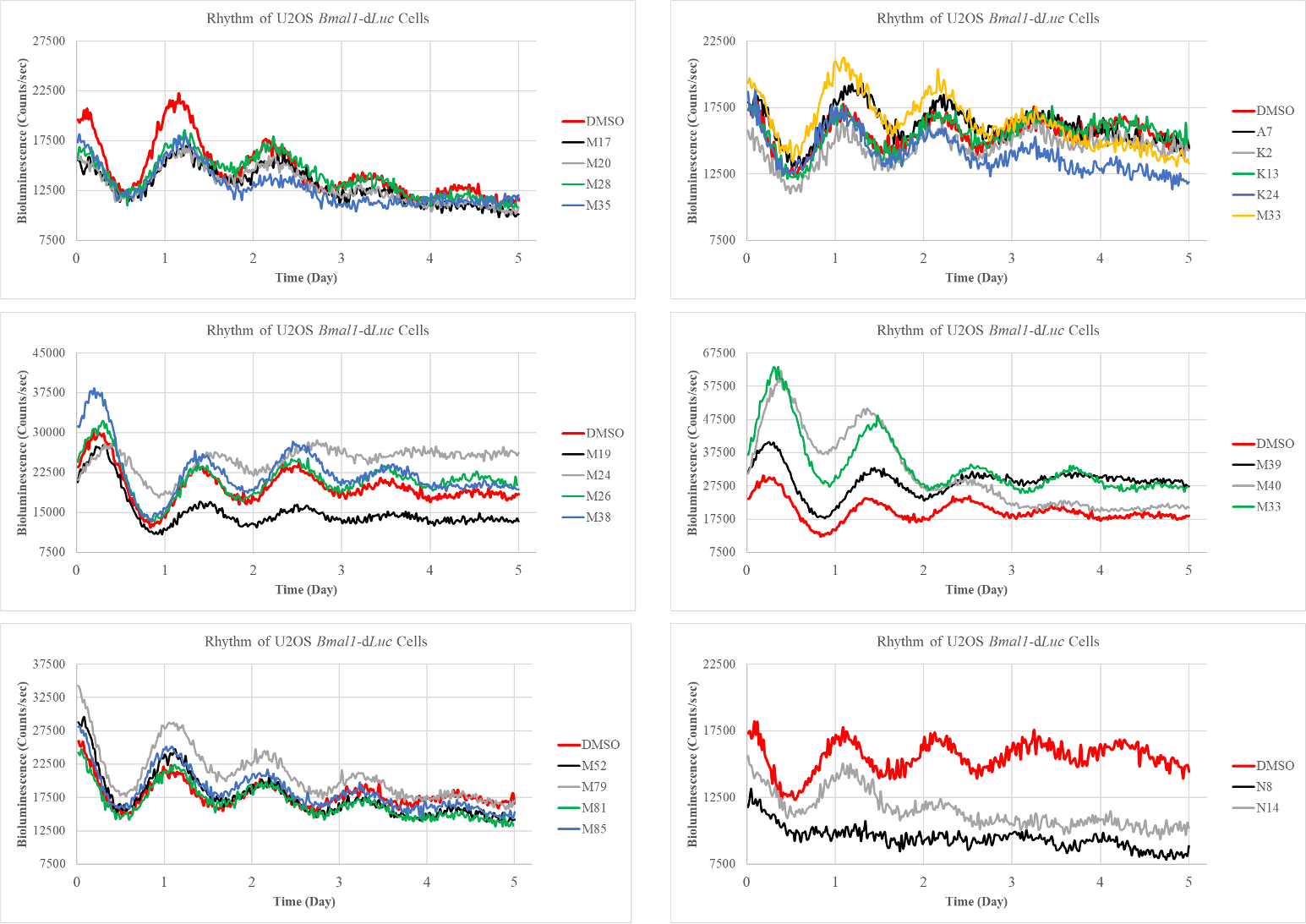


**Figure S3**: The effect of the molecules on circadian rhythm of U2OS cells.


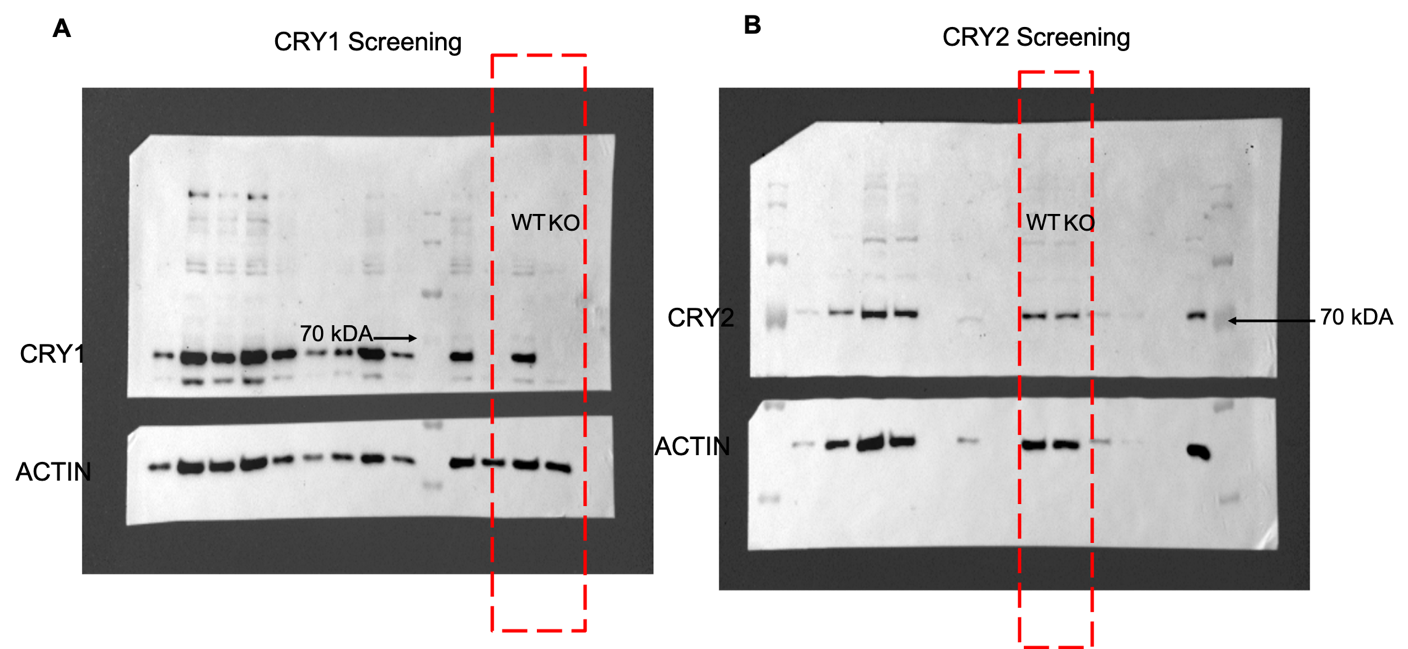


**Figure S4**: Confirmation of CRY1 knockout in U2OS cell line in Figure 4. (a) Immunoblot of CRY1 and (b) CRY2 proteins in the U2OS clone confirmed that specific knockout of CRY1 was successful. Actin was blotted as the loading control. Numbers and marks on the left of each panel indicate the positions of the corresponding molecular size markers in kDa. WT: Wildtype; KO: knockout.


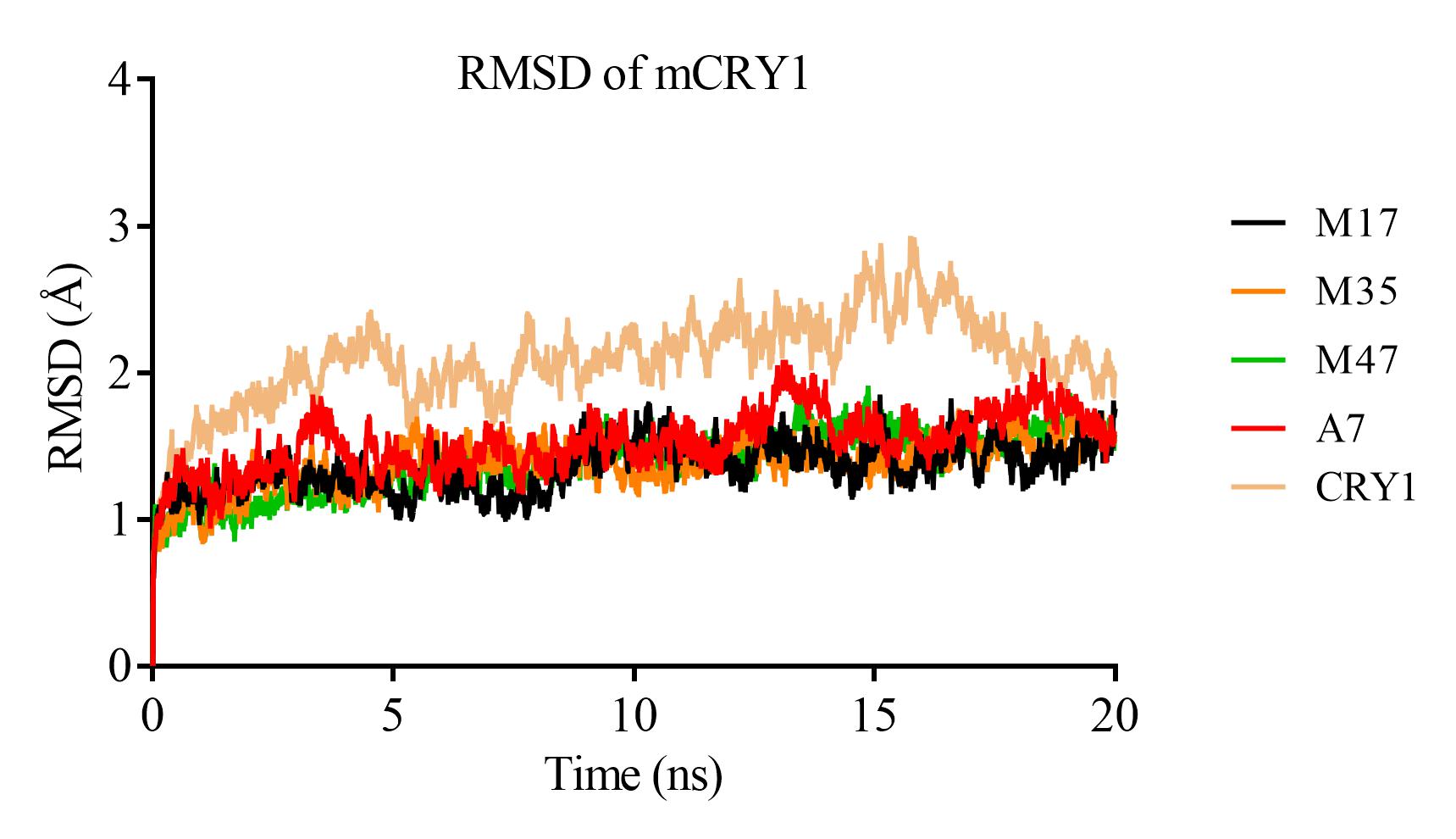


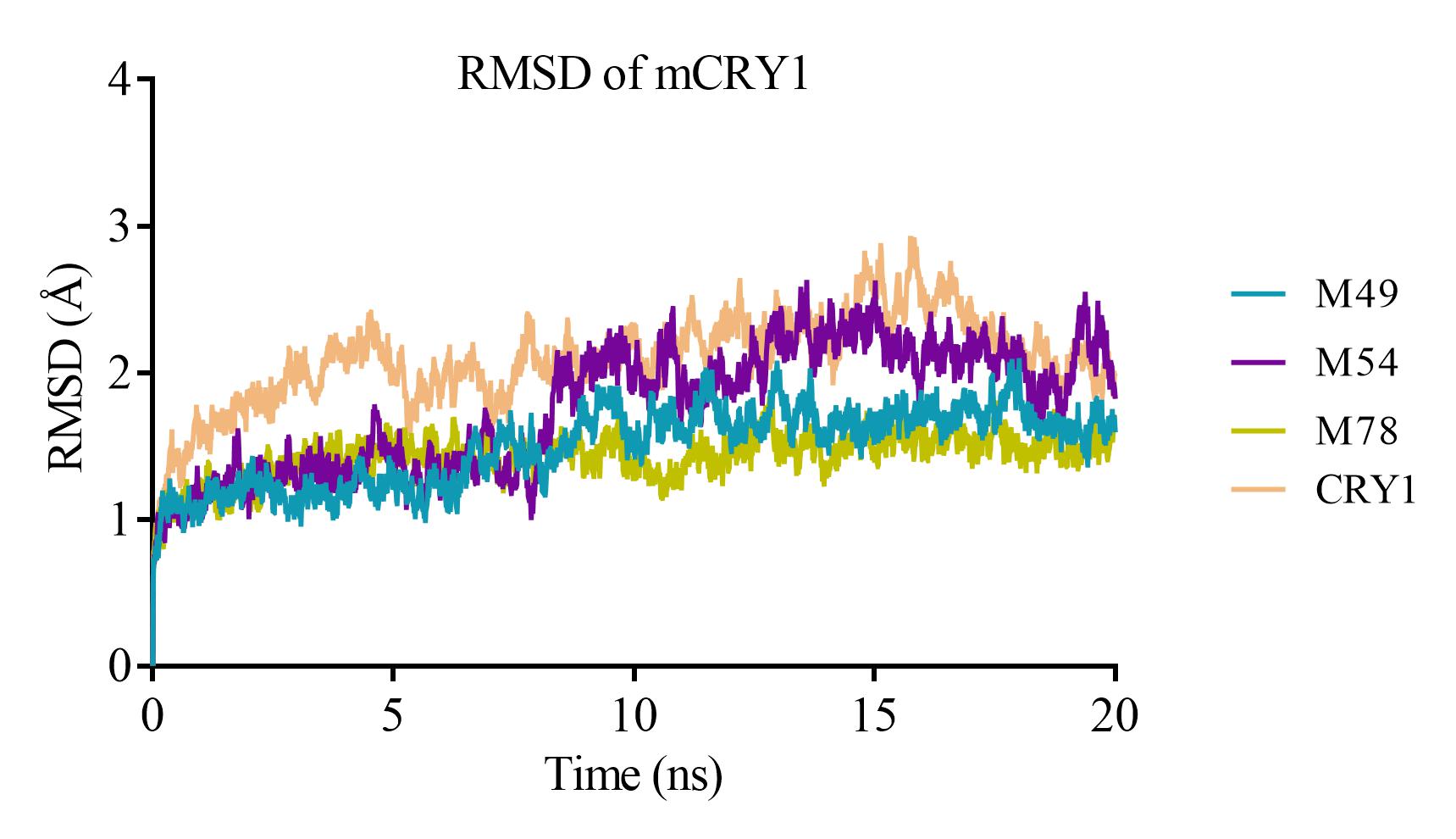


**Figure S5**: RMSD of CRY1backbone atoms with and without the simulation of molecules.

**Table S1**: Vina binding free energies of the molecules to CRY1.

**Table S2:** Toxicity Dataset, maximum of 10-Fold CV accuracies with 100 repetitions for feature sets with cardinality ranging from 2 to 20. DTC, RFC, ETC, and XGBC trained and tested on feature sets with cardinality between 2 and 20.

**Table S3:** Toxicity Dataset, standard deviation of 10-Fold CV accuracies with 100 repetitions for feature sets with cardinality ranging from 2 to 20. DTC, RFC, ETC, and XGBC trained and tested on feature sets with cardinality between 2 and 20.

**Table S4**: The period length of the U2OS *Bmal1*-d*Luc* cells treated with indicated molecules. Analyses were performed by using Biodare website.

**Table S5:** Period Changer Dataset, maximum of 10-Fold CV accuracies with 100 repetitions for feature sets with cardinality ranging from 2 to 20. DTC, RFC, ETC, and XGBC trained and tested on feature sets with cardinality between 2 and 20.

**Table S6:** Period Changer Dataset, standard deviation of of 10-Fold CV accuracies with 100 repetitions for feature sets with cardinality ranging from 2 to 20. DTC, RFC, ETC, and XGBC trained and tested on feature sets with cardinality between 2 and 20.
